# High discontinuation rate of azathioprine in autoimmune hepatitis, independent of time of treatment initiation

**DOI:** 10.1111/liv.14513

**Published:** 2020-06-11

**Authors:** Simon Pape, Tom J. G. Gevers, Jan Maarten Vrolijk, Bart van Hoek, Gerd Bouma, Carin M. J. van Nieuwkerk, Richard Taubert, Elmar Jaeckel, Michael P. Manns, Maria Papp, Nora Sipeki, Felix Stickel, Cumali Efe, Ersan Ozaslan, Tugrul Purnak, Frederik Nevens, Dominik J. N. Kessener, Alisan Kahraman, Heiner Wedemeyer, Johannes Hartl, Christoph Schramm, Ansgar W. Lohse, Michael A. Heneghan, Joost P. H. Drenth

**Affiliations:** ^1^ Department of Gastroenterology and Hepatology Radboud University Medical Center Nijmegen The Netherlands; ^2^ Department of Gastroenterology and Hepatology Rijnstate Hospital Arnhem The Netherlands; ^3^ Department of Gastroenterology and Hepatology Leiden University Medical Center Leiden The Netherlands; ^4^ Department of Gastroenterology and Hepatology VU University Medical Center Amsterdam The Netherlands; ^5^ Department of Gastroenterology, Hepatology and Endocrinology Hannover Medical School Hannover Germany; ^6^ Division of Gastroenterology Department of Internal Medicine Faculty of Medicine University of Debrecen Debrecen Hungary; ^7^ Department of Gastroenterology and Hepatology University Hospital of Zurich Zurich Switzerland; ^8^ Department of Gastroenterology Harran University Hospital Urfa Turkey; ^9^ Department of Gastroenterology Numune Research and Education Hospital Ankara Turkey; ^10^ Department of Gastroenterology Hacettepe University Ankara Turkey; ^11^ Department of Gastroenterology and Hepatology University Hospital KU Leuven Leuven Belgium; ^12^ Department of Gastroenterology and Hepatology University Clinic of Essen Duisburg Essen Germany; ^13^ 1st Department of Internal Medicine University Medical Center Hamburg‐Eppendorf Hamburg Germany; ^14^ Martin Zeitz Centre for Rare Diseases University Medical Centre Hamburg‐Eppendorf Hamburg Germany; ^15^ Institute of Liver Studies King's College Hospital London UK; ^16^ European Reference Network RARE‐LIVER Hamburg Germany

**Keywords:** autoimmune hepatitis, azathioprine, cohort study, immunosupression

## Abstract

**Background:**

Guidelines regarding treatment for autoimmune hepatitis (AIH) favour two strategies for azathioprine (AZA) introduction: concurrent with steroids at induction or delayed by 2‐4 weeks. The safety and efficacy of both strategies have been unexplored.

**Methods:**

We established a cohort of 900 AIH patients from 12 centres in 7 European countries. There were 631 patients who used AZA as part of the therapeutic regimen. We distinguished two groups: patients with early AZA (<2 weeks) or delayed AZA initiation (≥2 weeks). Primary outcome was discontinuation of AZA in the first year of treatment. Cox regression and propensity score matching was performed to determine difference in outcomes between groups.

**Results:**

Patients with early AZA initiation had significantly lower transaminases and bilirubin at baseline. Discontinuation rates of AZA did not differ between early and delayed starters (16.6% vs 14.2%), which did not reach statistical significance (hazard ratio 0.97, 95% confidence interval 0.61‐1.55, *P* = .90). Stratification according to baseline disease activity or propensity score matching did not alter the results. Main reason for AZA discontinuation was intolerance to treatment (14.0% vs 13.2%, *P* = .78) with nausea and vomiting as main side effects. AIH remission rates were comparable among groups.

**Conclusion:**

The discontinuation rate of AZA in AIH treatment is ~15% in the first year of treatment. Early or delayed AZA initiation does not differ in remission and discontinuation rates in AIH induction therapy. Our data suggest that either strategy may be used as part of AIH treatment.

**Key points**

- Discontinuation rate and efficacy of therapy for AIH in the first year of treatment is independent of time of AZA initiation.
- AZA discontinuation occurs in 15% of AIH patients in the first year.
- Discontinuation rates do not differ between patients who start AZA concurrent to steroid treatment and those who start AZA ≥2 weeks later.
- Most common side effects of AZA therapy in AIH are nausea, emesis en diarrhoea.

## 1 INTRODUCTION

Cornerstone of autoimmune hepatitis (AIH) treatment is steroid induction therapy, consisting of predniso(lo)ne, and subsequent addition of azathioprine (AZA).^1^ The use of AZA as therapy for AIH was first described anecdotally in the 1960s,^2^ and controlled studies over the next decades heralded the use of AZA as first‐line maintenance therapy in AIH.^3^, ^4^, ^5^ One study with a long follow‐up found that AIH patients are able to withdraw from steroids when on AZA therapy, while maintaining remission.^6^ Therefore, maintenance therapy with AZA is considered the mainstay treatment for AIH. While the vast majority of patients tolerate AZA therapy, approximately 10%‐20% of patients may develop side effects ranging from mild nausea to severe cytopenia or cholestatic hepatitis, which may lead to discontinuation of the drug. The exact incidence of AZA‐related side effects in AIH remains largely unknown since current evidence is based on trials that were conducted decades ago.^7^

There is ambiguity when to initiate AZA therapy after AIH onset. The American Association of Study of Liver Diseases (AASLD) guideline suggests to start AZA simultaneously with predniso(lo)ne,^8^ whereas the European Association for Study of the Liver (EASL) clinical practice guideline suggests that introduction of AZA could be delayed by 2 or more weeks. This would help to resolve possible diagnostic uncertainties and to discriminate between AZA‐induced hepatotoxicity and primary non‐response.^9^ Formal evidence that supports either strategy is absent from the literature. Using data from a large multicenter international retrospective cohort, we aimed to investigate the role of early (<2 weeks) vs delayed (≥2 weeks) introduction of AZA therapy and its effect on discontinuation rates, side effects of therapy and efficacy.

## 2 METHODS

### 2.1 Study design

We performed a retrospective cohort study with AIH patients from 12 centres in 7 countries in Europe. Patients were included if they were ≥18 years old and had a ‘probable’ or ‘definite’ AIH diagnosis according to the simplified diagnostic criteria established by the International Autoimmune Hepatitis Group.^10^ Only patients who used AZA as first‐line treatment and who started AZA within the first 26 weeks after diagnosis were included in this study. Patients with signs of variant syndromes with primary biliary cholangitis or primary sclerosing cholangitis were excluded. Patients with other forms of liver disease, such as histologically proven non‐alcoholic steatohepatitis or viral hepatitis, were excluded as well. Ethics approval was waived after review by local institutional review board.

### 2.2 Data collection

We collected patient data from original patient records and local databases. Demographical, serological, histological, biochemical and treatment data were collected using a predefined electronic case report form and stored in an online database (Castor Electronic Data Capture, CIWIT BV, Amsterdam, The Netherlands). In patients who had stopped AZA therapy, additional data were collected regarding reason of cessation of AZA therapy, duration of AZA use and switch to second‐line therapy.

### 2.3 Outcomes

Primary outcome was percentage of patients who discontinued AZA within the first year of treatment. Patients who had discontinued AZA therapy were classified as: (a) stopped as a result of intolerance to treatment or (b) stopped because of an insufficient response to treatment, as assessed by the treating physician. These two outcome measures were also analysed as separate endpoints. Adverse events that led to discontinuation of AZA therapy were collected from the patient record and included nausea, emesis, diarrhoea, rash, cytopenia, infection, pancreatitis, hepatitis, fever, arthralgia/myalgia and skin abnormalities. Secondary outcomes were serum transaminases below the upper limit of normal (ULN) after 26 weeks and 52 weeks of treatment. Both alanine aminotransferase (ALT) and aspartate aminotransferase (AST) needed to be below the ULN in order to meet this endpoint. In case an ALT or AST value was missing, the known ALT or AST value had to be below the ULN in order to meet this outcome. We used the gender‐specific ULN of each centre for ALT and AST. In addition, biochemical remission was a secondary outcome in the subgroup of patients with measured immunoglobulin G (IgG), which was defined a normalization of serum transaminases and IgG.

### 2.4 Analysis

Based on the initial start date of AZA, patients were divided into two groups: patients who had received AZA therapy within 2 weeks of initiation of steroid therapy (‘early’ AZA therapy) and patients who had received AZA 2‐24 weeks after steroid treatment (‘delayed’ AZA therapy). The choice between either therapeutic strategies was left at the discretion of the attending physician. Univariate comparisons between the groups were made using the chi‐square, Mann‐Whitney *U* or *t* test as appropriate. We used multivariable Cox regression, with the early AZA starters as group of interest, to determine differences in primary outcome between the two groups and to correct for possible confounders with time until AZA discontinuation as time‐dependent variable. We predefined a number of possible confounders that could have an association with the primary outcome. These included age, institute, gender and presence of cirrhosis. Significant baseline differences between the two groups were also added to the multivariable regression model. Results of the Cox regression are presented as hazard ratio's (HR) with 95% confidence intervals (CI) with the early AZA starts as group of interest. We performed multivariable logistic regression for the endpoints of normalization of transaminases and biochemical remission at weeks 26 and 52. Results of the logistic regression are presented as odds ratio's (OR) with 95% CI's. In addition, we used propensity score matching to compare matched groups of patients based on baseline disease activity. We used baseline values of transaminases and bilirubin to calculate a propensity score with early or delayed AZA initiation as dependent variable. Cases were matched 1:1 using nearest‐neighbour matching. Additionally, we performed a subgroup analysis in all patients, stratified according to baseline disease activity, based on AST level. Patients were divided into three equal groups based on their baseline AST, in order to detect differences in our primary outcome. A *P*‐value < .05 was considered statistically significant. Analyses were conducted with SPSS version 25 (IBM Corporation, Armonk, NY, USA).

## 3 RESULTS

### 3.1 Population

Our original cohort consisted of 900 AIH patients. Some 244 patients were excluded because either they did not use AZA as part of maintenance therapy or because of missing data. Another 25 patients were excluded because AZA was started >26 weeks after start of steroid induction therapy. Our final cohort consisted of 631 patients. A total of 229 patients (36.3%) started AZA concurrent with steroid induction therapy, while 402 patients (63.7%) started AZA only 1‐24 weeks after induction. Most patients (74.5%) were woman and mean age at diagnosis was 48.7 years old (standard deviation 16.6 years). Patients with early AZA initiation had lower biochemical disease activity at baseline: median ALT (5.13 × ULN vs 14.51 × ULN, *P* < .001), AST (3.73 × ULN vs 14.26 × ULN, *P* < .001) and bilirubin (22.0 vs 50.5 µmol/L, *P* < .001) were all lower when compared to patients with a delayed introduction of AZA (Table 1). Median IgG (data available for 541 cases) was also significantly lower in patients with early AZA therapy (18.0 vs 21.8 g/L, *P* < .001). The presence of cirrhosis at index biopsy was equally distributed between both groups (19.2% vs 16.7%, *P* = .42), while acute‐severe AIH (AS‐AIH, defined as absence of cirrhosis and an INR > 1.5^11^) occurred more frequently in the group with delayed AZA introduction (5.7% vs 13.9%, *P* < .001). Patients with early AZA were given lower initial predniso(lo)ne dosages (0.58 vs 0.62 mg/kg/day, *P* = .02) and higher initial AZA dosages (0.86 mg/kg/day vs 0.80 mg/kg/day, *P* = .02).

**TABLE 1:** Baseline and treatment characteristics of patients who started AZA therapy concurrent with steroid treatment vs patients with a delayed introduction of AZA

	Early AZA initiation n = 229	Delayed AZA initiation n = 402	*P*‐value
Female gender, n (%)	163 (71.2%)	307 (76.4%)	.15
Age at diagnosis, y (SD)	50.00 (16.51)	47.87 (16.58)	.12
Probable AIH, n (%)	100 (43.7%)	157 (39.1%)	.26
Definite AIH, n (%)	129 (56.3%)	245 (60.9%)	.26
ALT × ULN, median (IQR)	5.13 (14.51)	14.51 (24.43)	<.001
AST × ULN, median (IQR)	3.73 (15.24)	14.26 (24.39)	<.001
Bilirubin (µmol/L), median (IQR)	22.0 (56.0)	50.5 (155.8)	<.001
IgG (g/L), median (IQR)^a^	18.0 (9.3)	21.8 (12.1)	<.001
Cirrhosis, n (%)	44 (19.2%)	67 (16.7%)	.42
AS‐AIH, n (%)	13 (5.7%)	56 (13.9%)	.001
Median initial predniso(lo)ne dose, mg (IQR)	40 (35)	40 (30)	.02
Median initial predniso(lo)ne dose, mg/kg (IQR)	0.58 (0.57)	0.62 (0.47)	.02
Median initial AZA dose, mg (IQR)	50 (50)	50 (25)	.004
Median initial AZA dose, mg/kg (IQR)	0.86 (0.62)	0.80 (0.38)	.02

Abbreviations: AIH, autoimmune hepatitis; ALT, alanine aminotransferase; AS‐AIH, acute‐severe AIH; AST, aspartate aminotransferase; AZA, azathioprine; IgG, immunoglobulin G; IQR, interquartile range; SD, standard deviation; ULN, upper limit of normal.

^a^ Data available for 541 patients.

### 3.2 Discontinuation of AZA

Ninety‐five (15.1%) patients in the entire cohort discontinued AZA therapy in the first year of treatment. Discontinuation rates did not differ between early (<2 weeks) and delayed (≥2 weeks) AZA initiation (16.6% vs 14.2%, *P* = .42). Multivariable Cox regression with correction for institute, age, gender, cirrhosis, AST at baseline, initial predniso(lo)ne dose and initial AZA dose showed that there was no significant difference between groups of AZA discontinuation rates (corrected HR 0.97, 95% CI 0.61‐1.55, *P* = .90) (Table 2). In both groups most patients discontinued AZA due to intolerance to treatment (14.0% vs 13.2%, *P* = .78) (Table 3), which was not statistically significant after multivariable logistic regression (OR 0.41, 95% CI 0.08‐2.08, *P* = .41). Discontinuation rates as a result of insufficient response were similar among both groups (univariate analysis 2.6% vs 1.0%, *P* = .12, multivariate analysis OR 2.44, 95% CI 0.48‐12.39, *P* = .28).

**TABLE 2:** Results after multivariable Cox and logistic regression for patients with early initiation of AZA therapy

Cox regression	Uncorrected HR	*P*‐value	Corrected HR	*P*‐value
Discontinuation of AZA < 52 wk	1.09 (0.72‐1.64)	.69	0.97 (0.61‐1.55)^a^	.90

Logistic regression	Uncorrected OR	*P*‐value	Corrected OR	*P*‐value
Normal transaminases at week 24	0.85 (0.61‐1.19)	.34	0.74 (0.51‐1.06)^a^	.10
Biochemical remission at week 24	0.88 (0.59‐1.31)	.53	0.85 (0.55‐1.32)^a^	.47
Normal transaminases at week 52	0.93 (0.63‐1.36)	.69	0.75 (0.49‐1.15)^a^	.19
Biochemical remission at week 52	1.01 (0.64‐1.60)	.96	0.96 (0.57‐1.62)^a^	.87

Abbreviations: AZA, azathioprine; HR, hazard ratio; OR, odds ratio.

^a^ In all multivariable models we adjusted for institute, age, gender, cirrhosis, aspartate aminotransferase at baseline, predniso(lo)ne dose and AZA dose.

**TABLE 3:** Outcomes of patients who started AZA therapy concurrent with steroid treatment vs patients with a delayed introduction of AZA

	Early AZA initiation n = 229	Delayed AZA initiation n = 402	*P*‐value
Discontinued AZA < 52 wk, n (%)	38 (16.6%)	57 (14.2%)	.42
Intolerance	32 (14.0%)	53 (13.2%)	.78
Nausea	15 (6.6%)	28 (7.0%)	.84
Emesis	7 (3.1%)	13 (3.2%)	.90
Diarrhoea	2 (0.5%)	2 (0.9%)	.57
Rash	1 (0.2%)	1 (0.4%)	.69
Cytopenia	3 (1.3%)	8 (2.0%)	.53
Infection	0	1 (0.2%)	.45
Pancreatitis	3 (1.3%)	0	.02
Hepatitis	5 (2.2%)	10 (2.5%)	.81
Fever	0	2 (0.5%)	.29
Arthralgia/Myalgia	0	4 (1.0%)	.13
Skin abnormalities	0	1 (0.2%)	.45
Other^a^	0	7 (1.7%)	.045
Insufficient response	6 (2.6%)	4 (1.0%)	.12
Median duration of AZA use, wk (IQR)	6.0 (16.25)	6.0 (10.0)	.70
Switched to second‐line therapy < 52 wk, n (%)	31 (13.5%)	44 (10.9%)	.33
MMF	14 (6.1%)	13 (3.2%)	.09
6‐MP	12 (5.2%)	22 (5.5%)	.90
6‐TG	0	5 (1.2%)	.09
TAC	1 (0.4%)	1 (0.2%)	.69
CsA	4 (1.7%)	3 (0.7%)	.25
Normalization of transaminases at week 26, n (%)	129 (56.3%)	242 (60.2%)	.34
Biochemical remission at week 26, n (%)^b^	155 (57.5%)	83 (54.2%)	.53
Normalization of transaminases at week 52, n (%)^c^	121 (66.5%)	242 (68.2%)	.69
Biochemical remission at week 52, n (%)^d^	160 (66.4%)	84 (66.7%)	.96

Abbreviations: 6‐MP, 6‐mercaptopurine; 6‐TG, 6‐tioguanine; AZA, azathioprine; CsA, cyclosporine, IQR, interquartile range; MMF, mycophenolate mofetil; TAC, tacrolimus.

^a^ Hair loss, dizziness and headache.

^b^ Data available for 423 patients.

^c^ Data available for 537 patients.

^d^ Data available for 367 patients.

**FIGURE 1:**
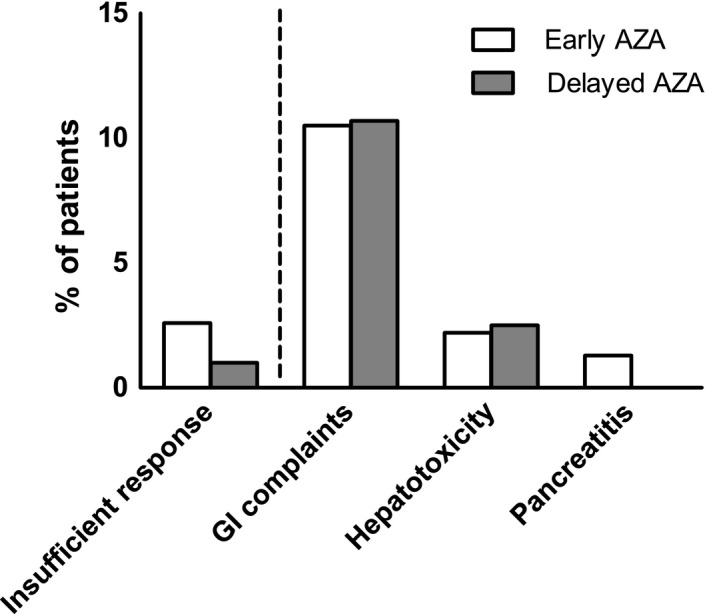
Rates of insufficient response and main side effects when patients experienced intolerance which lead to AZA discontinuation. AZA, azathioprine. GI, gastrointestinal

Most frequent reasons for stopping AZA resulted from gastrointestinal toxicity such as nausea (6.6% vs 7.0%, *P* = .84), emesis (3.1% vs 3.2%, *P* = .90) and hepatitis (2.2% vs 2.5%, *P* = .81) (Figure 1). Patients with gastrointestinal complaints did not receive higher initial AZA dosages than patients without gastrointestinal complaints (0.74 mg/kg vs 0.74 mg/kg, *P* = .87).

Three patients (1.3%) in the early AZA initiation group developed pancreatitis, while this did not occur in patients from the delayed AZA group (*P* = .02). Pancreatitis developed quickly after AZA initiation (median duration 2 weeks), while cytopenia only developed after a median duration of 32 weeks. Hepatitis related to AZA therapy developed after a median duration of 6 weeks (Figure S1; Table S1).

Our cohort consisted of 69 patients with AS‐AIH, of which the majority of patients (81.2%) belonged to the delayed AZA initiation group. Overall, AZA discontinuation rates in AS‐AIH were lower than in patients with ‘normal’ AIH (8.7%). Consequently, rates did not differ between early (1/13, 7.7%) and late (5/56, 8.9%) AZA initiation (*P* = .89).

### 3.3 Discontinuation rates after propensity score matching

Using propensity score matching, we established two matched groups of 146 patients in the early and delayed AZA initiation group. There were no significant baseline differences between the two groups. Consequently with our primary analysis, discontinuation rates of AZA therapy did not differ between early and delayed AZA initiation (16.4% vs 15.8%, *P* = .87) (Table 4). Additionally, there were no differences in rates of normalization of transaminases at week 24 and week 52 of treatment.

**TABLE 4:** Baseline characteristics and primary outcome after propensity score matching. A propensity score was created based on baseline disease activity. The matched cohort consisted of 292 patients

	Early AZA initiation n = 146	Delayed AZA initiation n = 146	*P*‐value
Baseline characteristics			
Female gender, n (%)	102 (69.9%)	114 (78.1%)	.11
Age at diagnosis, y (SD)	50.70 (16.67)	47.37 (16.18)	.08
ALT × ULN, median (IQR)	7.26 (17.32)	8.38 (14.33)	.49
AST × ULN, median (IQR)	6.94 (15.65)	6.80 (15.59)	.90
Bilirubin (µmol/L), median (IQR)	26.8 (66.3)	34.0 (102.0)	.24
IgG (g/L), median (IQR)^a^	19.31 (11.4)	20.74 (12.9)	.08
Cirrhosis, n (%)	30 (20.5%)	33 (22.6%)	.67
AS‐AIH, n (%)	8 (5.5%)	11 (7.5%)	.48
Median initial predniso(lo)ne dose, mg/kg (IQR)	0.60 (0.55)	0.57 (0.43)	.99
Median initial AZA dose, mg (IQR)	50 (50)	50 (25)	.17
Median initial AZA dose, mg/kg (IQR)	0.82 (0.49)	0.77 (0.36)	.35
Outcomes			
Discontinued AZA < 52 wk, n (%)	24 (16.4%)	23 (15.8%)	.87
Normal transaminases at week 24, n (%)	76 (52.1%)	84 (57.5%)	.35
Normal transaminases at week 52, n (%)^a^	78 (67.2%)	83 (64.3%)	.63

Abbreviations: ALT, alanine aminotransferase; AS‐AIH, acute‐severe autoimmune hepatitis; AST, aspartate aminotransferase; AZA, azathioprine; IgG, immunoglobulin G; IQR, interquartile range; SD, standard deviation; ULN, upper limit of normal.

^a^ Data available for 245 patients.

### 3.4 Treatment outcomes and switch to second‐line therapy

There were no differences regarding treatment outcomes between both study groups. Normalization of transaminases after 26 weeks of treatment was achieved by 56.3% of patients from the early AZA group compared to 60.2% of patients from the delayed AZA group (*P* = .34) (Table 2). After 52 weeks of treatment, rates of normalization of transaminases were similarly distributed (66.5% vs 68.2%, *P* = .69). When corrected for institute, age, gender, cirrhosis, AST at baseline, predniso(lo)ne dose and AZA dose, the OR for normalization of transaminases in early AZA starters was 0.74 (95% CI 0.51‐1.06, *P* = .10) after 24 weeks of treatment and 0.75 (0.49‐1.15, *P* = .19) after 52 weeks of treatment. In a subgroup of patients with available IgG data (n = 426 for week 26, n = 367 for week 52), rates of biochemical remission were not different between the two groups (week 26: 57.5% vs 54.2%, *P* = .53; week 52: 66.4% vs 66.7%, *P* = .96). Corrected ORs for biochemical remission in patients with early AZA initiation were 0.85 (95% CI 0.55‐1.32, *P* = .47) for 24 weeks and 0.96 (95% CI 0.57‐1.62, *P* = .87) for 52 weeks. Most patients switched to second‐line therapy shortly after AZA was stopped: 81.6% of patients in the early AZA group vs 77.2% of patients in the delayed group (*P* = .33). Drugs patients most frequently switched to were 6‐mercaptopurine and mycophenolate mofetil (Table 2).

### 3.5 Stratification according to baseline disease activity

When stratified according to baseline disease activity, we did not find any significant differences for discontinuation rates of AZA between the three groups (Table 5). Additionally, occurrence of AZA‐induced hepatotoxicity, rates of normalization of transaminases and biochemical remission at week 52 were not statistically different between the three groups.

**TABLE 5:** Rates of AZA discontinuation < 52 wk stratified according to baseline AST levels

	Early AZA initiation	Delayed AZA initiation	*P*‐value
AST baseline 0.03‐4.8 × ULN			
Discontinued AZA < 52 wk, n (%)	24 (19.5%)	20 (23.0%)	.54
Normal TA week 52	58 (63.7%)	47 (63.5%)	.98
Biochemical remission week 52 N = 116	39 (60.0%)	30 (58.8%)	.90
Hepatotoxicity	2 (1.6%)	2 (2.3%)	.73
AST baseline 4.9‐19.4 × ULN			
Discontinued AZA < 52 wk, n (%)	9 (15.5%)	22 (14.9%)	.91
Normal TA week 52	28 (60.9%)	89 (66.9%)	.46
Biochemical remission week 52 N = 128	22 (71.0%)	60 (61.9%)	.36
Hepatotoxicity	1 (1.7%)	6 (4.1%)	.41
AST baseline 19.5‐136 × ULN at baseline			
Discontinued AZA < 52 wk, n (%)	5 (11.1%)	15 (9.0%)	.67
Normal TA week 52	34 (79.1%)	106 (72.1%)	.36
Biochemical remission week 52 N = 121	23 (82.1%)	70 (75.3%)	.45
Hepatotoxicity	2 (4.4%)	2 (1.2%)	.16

Abbreviations: AST, aspartate aminotransferase; AZA, azathioprine; TA, transaminases; ULN, upper limit of normal.

## 4 DISCUSSION

Our study shows that discontinuation rate and efficacy of medical therapy for AIH in the first year of treatment is independent of time of AZA initiation. AZA discontinuation occurred in ~15% of our cohort. The exact number of AZA discontinuation rates in AIH is unknown and guidelines usually refer to the first trials investigating AZA in AIH, which lack comparison to real‐world practice.^7^, ^8^ Real‐world data on AZA discontinuation in AIH are limited: one study found a discontinuation rate of 6.8%, which is lower than the number we report in this study.^12^ Interestingly, we found that discontinuation rates in patients with AS‐AIH are lower than in patients with non‐severe AIH. The reason for this is unknown but probably relates to patient‐ and physician‐related factors. We hypothesize that if patients present with severe AIH, physicians will apply a more stringent approach towards treatment optimization and clinical follow‐up, resulting in better treatment outcomes.

Discontinuation rates did not differ between patients who started AZA concurrent to steroid treatment and those who started AZA ≥2 weeks later. Additionally, we found no differences in rates of normalization of transaminases and biochemical remission between patients with early or delayed AZA initiation. The strategy of delaying introduction of AZA therapy in order to avoid hepatoxicity in the early stages of the disease has found its way to guidelines, but evidence supporting this strategy is absent.^9^, ^13^

Data regarding the specific AZA‐related side effects in AIH also originate from the first AIH trials or are derived from other auto‐inflammatory diseases such as ulcerative colitis or rheumatoid arthritis.^14^, ^15^ One single centre study found that 5% of AIH patients discontinued AZA therapy as a result of side effects within 1 month of AZA initiation, compared to 29% of patients with Crohn's disease.^16^ We report similar rates and found that 13.4% of patients discontinued AZA owing to side effects, and 5.7% did so within the first month. We found that acute pancreatitis related to AZA therapy is more likely to occur in the first weeks after initiation, while cytopenia may occur up to 40 weeks after initiation of treatment. These findings provide a better understanding of the temporal dynamics between type of and duration of AZA‐related adverse events. We observed that the gastrointestinal toxicity such as nausea, emesis and diarrhoea occurs most frequently in this population. We did not find a relationship between AZA dose and occurrence of gastrointestinal complaints, suggesting that gastrointestinal toxicity caused by AZA use is an idiosyncratic reaction.

Our study shows that patients with a delayed AZA introduction are more likely to have high transaminases at baseline, which suggests that some physicians had been reluctant to start AZA in these patients. However, when stratified according to baseline disease activity, we found that there were no differences in discontinuation rates between early or delayed starters of AZA, indicating that timing of introduction did not exert a profound effect on this parameter. Furthermore, there were no differences regarding normalization of transaminases and biochemical remission between early or delayed introduction of AZA, suggesting that delayed introduction does not impair treatment efficacy. We observed a statically significant difference in initial AZA dose between the early and delayed groups. Patients with early AZA initiation were treated with slightly higher AZA dosages than patients with delayed AZA initiation, suggesting that higher AZA dosages could have influenced our results. However, we argue that the difference of 0.06 mg/kg (translating to ~4 mg AZA in a 70 kg patient) is of little clinical relevance. Furthermore, we corrected for AZA dose in our multivariate analyses.

Our study has some inherent limitations. First, because of the retrospective design of our study, there is a risk of selection bias and confounding by indication. Only patients with sufficient data were included in this study, limiting the generalizability. This is, however, the largest multicenter study to provide a real‐world insight in reasons for stopping AZA therapy in AIH treatment. Second, we used assessment from the treating physician to define AZA‐related side effects, instead of a priori standardized definitions, which would be difficult to assess owing to possible under‐reporting in patients' records. We chose discontinuation of AZA as our primary endpoint because, in case of AZA intolerance or insufficient response, stopping of AZA will be the likely consequence. Third, we do not provide any data on use of 6‐thioguanine nucleotide levels (6‐TGN) for treatment monitoring in AIH. Although there may be a clinical benefit in therapeutic drug monitoring of AIH patients while on thiopurine therapy,^17^, ^18^, ^19^ we found that the practice of measuring 6‐TGN levels was not broadly established. Fourth, we do not provide data on the long‐term risk of AZA use. It is known that long‐term use of immunosuppressants including AZA increases the risk of malignancies, particularly non‐melanoma skin cancer, and lymphoproliferative disorders.^20^, ^21^, ^22^ However, it is unlikely that these long‐term risks will be influenced by early or delayed initiation of AZA.

The data from our study support both strategies of immediate AZA initiation (AASLD) or delayed AZA introduction (EASL), as laid out in international guidelines. The main reason to delay AZA introduction is to discriminate between AZA‐induced hepatotoxicity and non‐response to treatment. However, we found that AZA‐induced hepatitis was uncommon and occurred in both groups regardless of baseline disease activity, and that AZA‐induced hepatotoxicity developed only after a median duration of 6 weeks of treatment. This suggests that delayed AZA introduction to avoid this side effect is not that useful.

In conclusion, the discontinuation rate of AZA in AIH patients is ~15% in the first year of treatment. Early or delayed AZA initiation does not differ in remission and discontinuation rates in AIH induction therapy. Our data suggest that either strategy may be used as part of AIH treatment.

## CONFLICT OF INTEREST

None declared.

## Supporting information

Fig S1

Table S1
