# Supplementary figures and images for: High discontinuation rate of azathioprine in autoimmune hepatitis, independent of time of treatment initiation
Source: Liver Int. 2020 Jun 11;40(9):2164–71. doi: 10.1111/liv.14513 (PMC7496382; doi:10.1111/liv.14513)

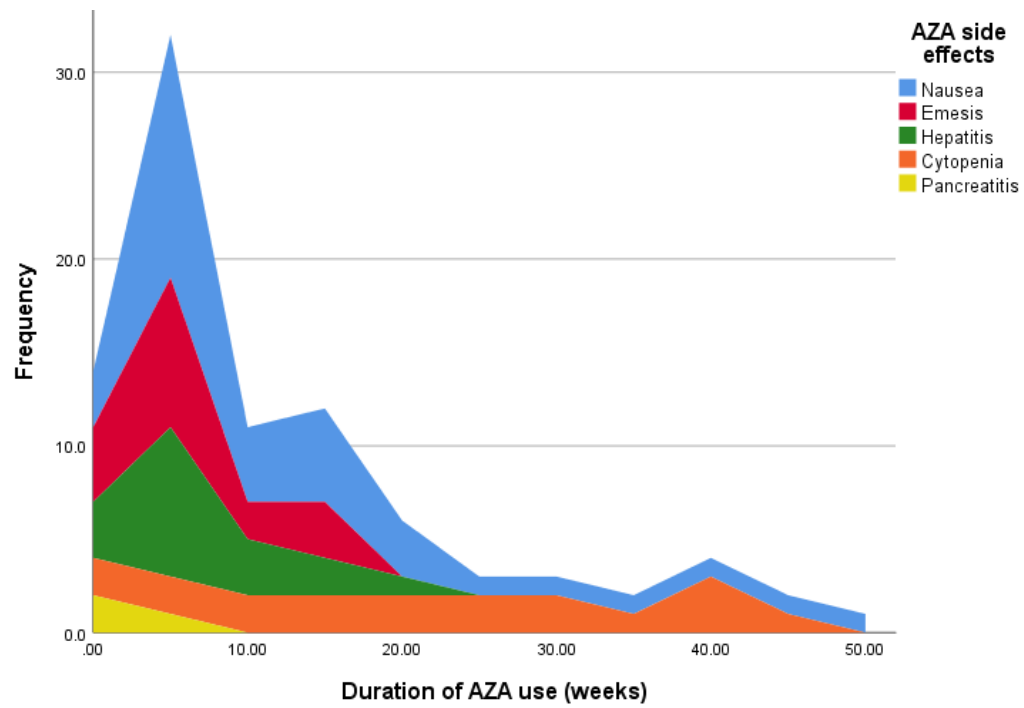

Supplement: Supplementary file 1 — Fig S1 [file LIV-40-2164-s001.pdf]
