# Supplementary material for: High discontinuation rate of azathioprine in autoimmune hepatitis, independent of time of treatment initiation
Source: Liver Int. 2020 Jun 11;40(9):2164–71. doi: 10.1111/liv.14513 (PMC7496382; doi:10.1111/liv.14513)
Supplement: Supplementary file 2 — Table S1 [file LIV-40-2164-s002.docx]

|  | **Occurrence in total cohort, n (%)** | **Median duration to occurrence, weeks (range)** |
| --- | --- | --- |
| Discontinuation of AZA due to intolerance | 85 (13.5%) | 6 (0 – 51) |
| Nausea | 43 (6.8%) | 6 (0 – 51) |
| Emesis | 20 (3.2%) | 4.5 (0 – 13) |
| Diarrhea | 4 (0.6%) | 5 (4 – 10) |
| Rash | 2 (0.3%) | 4.5 (1 – 8) |
| Cytopenia | 11 (1.7%) | 32 (2 – 45) |
| Infection | 1 (0.2%) | 34* |
| Pancreatitis | 3 (0.5%) | 2 (2 – 4) |
| Hepatitis | 15 (2.4%) | 6 (1 – 18) |
| Fever | 2 (0.3%) | 7 (3 – 11) |
| Arthralgia / Myalgia | 4 (0.6% | 10 (4 – 44) |
| Skin abnormalities | 1 (0.2%) | 22* |

**Supplementary table 1**: Occurrence and median duration towards manifestation of azathioprine related adverse events leading to discontinuation of azathioprine discontinuation in the first 52 weeks of treatment. * Numbers were too low to provide a range. Patients could have multiple side-effects that led to discontinuation. AZA, azathioprine.
